# Analysis of Characteristics of Endometrial Carcinoma in Peri- and Postmenopausal Women with Abnormal Uterine Bleeding

**DOI:** 10.1155/2024/6509171

**Published:** 2024-02-24

**Authors:** Ye Yanli, Wang Tian Mei, Li Cong

**Affiliations:** ^1^Department of GynecologyNanan People's Hospital of Chongqing, Chongqing 400060, China; ^2^Department of Gynecology, First Affiliated Hospital of Chongqing Medical University, Chongqing 400060, China

## Abstract

**Objective:**

To analyze the menstrual characteristics of endometrial carcinoma and investigate whether abnormal uterine bleeding in the perimenopausal period differs from postmenopausal bleeding.

**Methods:**

We conducted a retrospective analysis of 928 cases of endometrial carcinoma in patients admitted from January 2016 to December 2022. We gathered fundamental clinical data and analyzed distinct clinical risk factors between the perimenopausal and postmenopausal groups. Furthermore, we computed the statistical variances in menarche, regular menstrual cycles, and the duration of abnormal uterine bleeding.

**Results:**

Perimenopausal patients with endometrial carcinoma exhibit similar factors to postmenopausal patients, especially if they have a history of menstrual cycles lasting more than 30 years, hypertension, abnormal uterine bleeding for over 1 year, and a high risk of endometrial carcinoma. Early intervention for abnormal uterine bleeding during the perimenopausal stage can prevent up to 80% of women from developing endometrial carcinoma.

**Conclusion:**

Perimenopause women experiencing abnormal uterine bleeding should be mindful of the risk of endometrial carcinoma, as this awareness can substantially decrease the occurrence of the disease.

## 1. Introduction

Endometrial carcinoma is the most prevalent gynecological cancer in high-income countries, and its incidence is on the rise globally [1]. The most frequent clinical symptom is postmenopausal bleeding, and it is highly prevalent among perimenopausal women with abnormal uterine bleeding [2]. These symptoms are the primary clinical manifestations of endometrial carcinoma. As the prevalence continues to increase [3], two distinct bleeding patterns display different clinical characteristics, such as variations in stage characteristics and degrees of differentiation. However, there are no related reports available. In clinical practice, when addressing abnormal uterine bleeding, it is crucial to not only achieve hemostatic effects but also to rule out the possibility of endometrial cancer. Gynecological ultrasound also demonstrates outstanding performance in this diagnosis [4]. By assessing the thickness of the endometrium, the uniformity of the echo, and the blood flow resistance index, it may be possible to differentiate high-risk populations based on menstrual characteristics in clinical practice. This could significantly aid in the early diagnosis of endometrial lesions and the detection of endometrial cancer. In this study, we analyzed the disparities in risk factors for endometrial carcinoma between the two groups, as well as any variations in clinical stage and differentiation postonset. Additionally, we investigated the association between menopause and the onset of endometrial carcinoma. These differences may serve as effective clinical features for the early identification of endometrial carcinoma. Additionally, the aim is to analyze the high-risk factors of endometrial carcinoma based on changes in bleeding patterns.

## 2. Data and Methods

### 2.1. Patient Information

The clinical data of patients diagnosed with endometrial carcinoma and admitted to the First Affiliated Hospital of Chongqing Medical University from January 2016 to December 2022 were collected. A total of 928 cases were divided into two groups based on the menopausal status of the patients. The clinical data of the included patients were retrospectively collected, and their clinical characteristics were analyzed and discussed. The clinical trial was approved by the hospital's ethics committee.

All patients were diagnosed with endometrial carcinoma through pathology following endometrial biopsy. After the comprehensive staging of endometrial carcinoma, the case data were updated according to the pathological staging of the surgery. The exclusion criteria are as follows: (a) patients with endometrium without atypical hyperplasia, endometrial serous adenocarcinoma, mucinous adenocarcinoma, clear cell carcinoma, and other special pathological types, as confirmed by pathological examination; (b) patients with functional ovarian malignant tumor, oral hormone therapy for breast cancer, and incomplete data; and (c) incomplete data collection.

### 2.2. Methods

#### 2.2.1. Data Design

The overall situation, family history, birth history, contraceptive methods, menstrual status, medical history, treatment history, and pathological data examined the statistical differences in the onset of menarche, regularity of menstrual cycles, and duration of abnormal uterine bleeding. If the menstrual cycle or duration of menstruation changes for more than 7 days, it is considered a menstrual anomaly. If a menstrual anomaly does not occur more than twice within a year, it is considered a regular menstrual cycle. According to the FIGO terminology in 2019 [4], abnormal uterine bleeding includes menstrual duration exceeding 7 days or less than 3 days, menstrual cycle longer than 45 days or less than 21 days, and menstrual volume exceeding 80 ml or less than 5 ml. A total of 928 patients with endometrial carcinoma were counted and recorded, and the database was established. The aim of this study is to compare the regular menstrual cycle length, menstrual characteristics, and time limit between the perimenopausal and postmenopausal groups.

#### 2.2.2. Statistical Methods for Data Analysis

The Shapiro-Wilk test is utilized to assess whether the variables adhere to a normal distribution using Excel software and SPSS 26.0 statistical software. Measurement data that follow a normal distribution are typically presented as mean ± standard deviation and analyzed using a *t*-test. For parameters that do not follow a normal distribution, the median and interquartile range are used, and the Mann–Whitney *U* test is applied. Count data are expressed as rates or percentages (%) and analyzed using the chi-square test. *P* value is used as a criterion for determining statistical significance. The Kaplan-Meier and Cox proportional hazards models were used for the survival analysis of endometrial carcinoma in Results. Survival analysis was conducted to determine if there was a significant difference in the time from abnormal uterine bleeding as the starting point to the diagnosis of endometrial carcinoma as the endpoint.

## 3. Results

### 3.1. Clinical Features of Abnormal Uterine Bleeding in Perimenopausal and Postmenopausal Populations

There were a total of 928 cases of endometrial carcinoma patients, divided into menopausal and onset groups. The sample sizes of the two groups were not significantly different. Data on age, BMI, pregnancy, delivery, contraception, hypertension, diabetes complications, tumor differentiation, and clinical stage were collected for each group. The data was then analyzed to determine if there were any statistical differences between the two groups (as shown in Table 1). Factors such as age of onset, age at menarche, pregnancy history, birth history, hypertension, diabetes, and the degree of disease differentiation should be considered. It is not related to BMI, contraceptive method, or menstrual cycle. The data indicates that a lower number of births before menopause and a later age at first menstruation are associated with a higher risk of endometrial carcinoma. Postmenopausal women are at a higher risk of developing endometrial carcinoma, particularly if they have hypertension and diabetes. Furthermore, the degree of differentiation of postmenopausal endometrial carcinoma tends to be worse compared to cases in perimenopause. It is important to note that there is no correlation between menopause and the stage of endometrial carcinoma.

In Table 1, analysis of several characteristics revealed the following findings: menarche, average menstruation duration of 30 years, and mean abnormal bleeding duration of more than 1 year after disease. When comparing the groups, it was observed that the perimenopausal group had a lower age at menarche and a shorter regular menstrual duration (30.55 ± 6.29 vs. 34.03 ± 6.45). Additionally, the duration of abnormal uterine bleeding was longer in the group compared to postmenopausal bleeding (14.87 ± 26.87 vs. 7.12 ± 12.07), exceeding the duration of menarche.

### 3.2. Survival Analysis of Endometrial Carcinoma in relation to Abnormal Uterine Bleeding

The onset of endometrial carcinoma is characterized by abnormal uterine bleeding, which acts as a critical point for analysis. Survival analysis was conducted to assess whether there was a significant difference in the duration of abnormal uterine bleeding as the starting point and the diagnosis of endometrial carcinoma as the endpoint and whether there was a statistical difference between the duration of abnormal uterine bleeding and the development of endometrial carcinoma. According to the menopausal status of the patients, the time limit for analyzing endometrial carcinoma patients was determined. The median survival time of the two groups was 2 months versus 4 months. The *P* value was less than 0.05, indicating a statistically significant difference. The average duration of bleeding time for the two groups was approximately six months.

**Figure 1:**
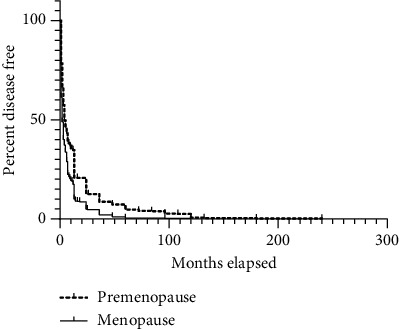
Vaginal bleeding duration in endometrial carcinoma patients with menopausal or not.

As depicted in Figure 1, the analysis of patient survival from abnormal uterine bleeding to the diagnosis of endometrial carcinoma revealed two distinct groups with statistically significant differences. Patients in the high differentiation group exceeded those in the postmenopausal group, while patients in the low differentiation group were fewer than those in the menopausal group. The degree of differentiation within each group did not show statistical differences, but there were significant variations between the groups.

**Figure 2:**
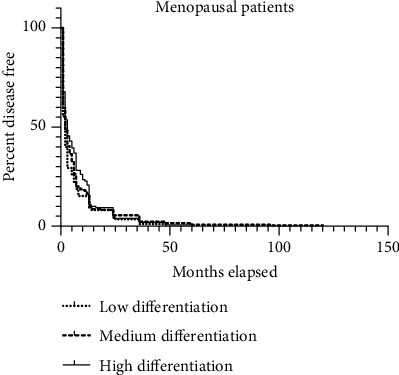
Duration of vaginal bleeding in different stages of postmenopause in patients with endometrial carcinoma.

In Figure 2, there was no statistically significant difference in the time at 1 month, 2 months, and 3 months, respectively, among postmenopausal patients with endometrial carcinoma of varying differentiation (*P* = 0.2329). The pairwise comparison yielded the following results: low-medium (0.3024), low-high (0.3188), and medium-high (0.0874).

**Figure 3:**
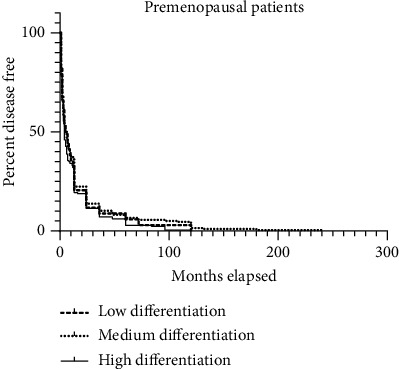
Abnormal uterine bleeding in different stages of perimenopause in patients with endometrial carcinoma.

In Figure 3, there was no statistically significant difference in the diagnosis of abnormal uterine bleeding at 6 months, 5 months, and 4 months among perimenopausal endometrial carcinoma patients (*P* = 0.2635). The pairwise comparison resulted in a value of 0.7650, with a minimum of 0.5528 and a maximum of 0.1071. However, it was also found that the median survival time for the low differentiation group was the longest compared to the postmenopausal group in Figure 2.

### 3.3. Analyze whether There Are Distinct Clinical Characteristics among Different Groups Based on the Duration of Abnormal Uterine Bleeding

**Figure 4:**
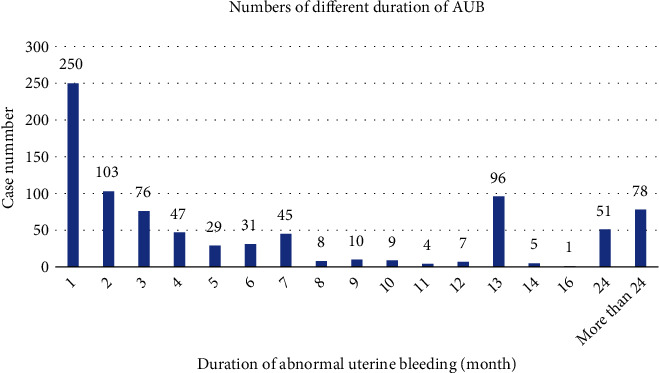
The numbers of different durations of abnormal uterine bleeding.

The duration of abnormal uterine bleeding was recorded over several months.  Figure 4 illustrates the number of patients with endometrial carcinoma at different durations of abnormal uterine bleeding. To ensure comparable patient number statistics, the study categorized the abnormal uterine bleeding time limit as follows: less than or equal to 1 month, 1-6 months, 6-24 months, and greater than 24 months.

According to the duration of abnormal uterine bleeding, we divided the cases into four groups to analyze the factors associated with endometrial carcinoma: bleeding duration of less than 1 month, 1-6 months, 6-24 months, and greater than 24 months. It was found that three clinical characteristics—age, menopause, and clinical stage—showed statistical differences, while menarche age, number of pregnancies and deliveries, and degree of differentiation showed no statistical differences. Patients with an onset time limit of less than or equal to 1 month were the oldest. Although the majority of patients were at stage I, the number of patients with a poorly differentiated pathological type was relatively high. Among perimenopausal patients, the largest proportion of abnormal uterine bleeding occurred between 7 and 24 months, accounting for 37.84% of all perimenopausal groups. Additionally, 80.30% of patients experienced persistent abnormal uterine bleeding for more than one month until diagnosis, while only 19.70% of patients were diagnosed with endometrial carcinoma within one month of experiencing abnormal uterine bleeding (see Table 2).

## 4. Discussion

### 4.1. Do Perimenopausal Endometrial Carcinoma Patients Exhibit Different Clinical Characteristics Compared to Postmenopausal Patients?

High-risk factors for cancer include genetic predisposition, early age at menarche, late age at menopause, a predisposition to hypertension, and obesity [5]. In particular, the incidence of endometrial recurrence rejuvenation is much higher in patients with polycystic ovary syndrome compared to the general population [6]. According to the results of this retrospective analysis, there were differences in the clinical characteristics of the two groups, including late menopause, early menarche, and fewer pregnant and reproductive patients. Postmenopausal bleeding is a prevalent symptom of endometrial carcinoma. When postmenopausal women experience bleeding, it is important to be vigilant. This clinical phenomenon has received significant attention. However, women in the perimenopausal stage may initially observe abnormal uterine bleeding instead of seeking immediate medical attention. A study report states that endometrial carcinoma is rare in perimenopausal women with abnormal uterine bleeding who undergo dilation and curettage (D&C) [7].

Age and BMI should not be considered when selecting patients. Several studies have shown that diabetic obese women with an endometrial thickness (ET) greater than 11 mm who experience perimenopausal vaginal bleeding have a 25% higher risk of premalignant/malignant endometrial pathology [8, 9]. Another study indicated that perimenopausal women over 40 years of age, with hypothyroidism, a BMI over 25 kg/m^2^, and a thickened endometrium (thicker than 13 mm) are at a high risk of EH/EC [10]. Therefore, it is recommended to consider an endometrial biopsy in these cases.

Perimenopausal patients have a lower risk of hypertension and diabetes compared to menopausal patients [11]. However, there were no statistically significant differences in terms of BMI, family history of cancer, and contraception between the two groups. This indicates that obese patients with a family history of cancer are at risk of developing endometrial carcinoma if they experience abnormal uterine bleeding, irrespective of their menopausal status.

### 4.2. Is the Clinical Stage and Differentiation of Perimenopausal Endometrial Carcinoma Patients Better than Those of Postmenopausal Patients?

To investigate the time limit for abnormal uterine bleeding as a predictor for the occurrence of endometrial carcinoma, we analyzed the duration of abnormal uterine bleeding in perimenopausal women. We compared the incidence of endometrial carcinoma in women with abnormal uterine bleeding of varying durations to that in the postmenopausal group. Statistical analysis was conducted to identify any significant differences in the duration of abnormal uterine bleeding between perimenopausal women who had a typical menstrual cycle of 30 days and those who experienced abnormal uterine bleeding for more than six months. Compared to the 35-year menstrual cycle in the menopausal group, abnormal uterine bleeding occurred for 14 months. This indicates that perimenopausal patients have a higher risk of abnormal uterine bleeding within a certain time frame compared to postmenopausal women, especially when their regular menstrual period continues beyond 36-year menstrual cycles. This is consistent with clinical observations that late menopause and reduced fertility are significant risk factors for endometrial carcinoma.

Therefore, perimenopausal women should pay attention to abnormal uterine bleeding. Segmental curettage is an important and effective method for eliminating endometrial carcinoma [12]. An analysis of perimenopausal women with endometrial carcinoma revealed that over 80% of patients experiencing abnormal uterine bleeding did not seek medical attention. Additionally, 37.84% of patients with abnormal uterine bleeding experienced symptoms for more than six months. Early detection and intervention, including prompt pathological biopsy, can help prevent the development of the disease in 80% of patients.

### 4.3. Is the Risk of Persistent Abnormal Uterine Bleeding Lower in Premenopausal Patients with Endometrial Carcinoma Compared to Menopausal Patients?

Are postmenopausal patients with endometrial carcinoma at a higher risk of developing a malignant tumor and experiencing a negative impact on their survival and prognosis? The retrospective study analyzed the clinical stage and degree of differentiation to determine the effective survival of endometrial carcinoma [13]. The analysis of two groups of endometrial carcinoma patients, divided by menopausal status, found no significant clinical difference in the diagnosis of endometrial carcinoma between perimenopausal and postmenopausal patients. However, there were differences in the proportion of differentiation degree. Perimenopausal endometrial carcinoma patients had a higher proportion of high differentiation compared to the postmenopausal group, while the number of patients with low differentiation was lower in the perimenopausal group compared to the postmenopausal group. Once again, a clinical reminder: Early diagnosis is crucial for abnormal uterine bleeding in perimenopausal women, even if the patient has developed cancer. High differentiation in cancer cells is associated with a significantly increased incidence [14]. A study has found that histopathological results, hemoglobin concentration, and basic sonographic parameters should be combined in evaluating intrauterine abnormalities in women with perimenopausal and postmenopausal bleeding [15]. For changes in abnormal uterine bleeding, combined with ultrasound imaging, screening for high-risk populations will also have good prospects. We will incorporate statistical analysis of bleeding volume in our future prospective studies.

The molecular classification of endometrial carcinoma predicts prognosis and is integrated into clinical stages and guidelines [16, 17]. Retrospective analysis has also sought to examine the relationship between the four molecular classifications and the duration of abnormal uterine bleeding. However, due to the small sample size in previous studies on the detection of endometrial carcinoma molecular classification, only 41 patients had a complete molecular classification report. The small sample size prevented the detection of any differences within the abnormal uterine bleeding time limit, and therefore no significant findings were observed. In future clinical observation experiments, the mentioned clinical characteristics will continue to be gathered. This will enable a preliminary prediction of the occurrence and prognosis of endometrial carcinoma, with a specific focus on the most common abnormal clinical manifestations of uterine bleeding. Additionally, it will provide clinical data support for the early diagnosis and treatment of endometrial carcinoma [18, 19]. Due to the retrospective nature of this study, no accurate data on the duration of menopause and current computerized bleeding were collected. Future analyses will focus on this aspect.

## 5. Conclusion

Although postmenopausal bleeding is a common symptom of endometrial carcinoma, there are similar risk factors between perimenopause and postmenopause. Specifically, diabetic patients with abnormal uterine bleeding who have had a regular menstrual cycle for more than 30 years are at a higher risk of developing endometrial carcinoma. The diagnosis of endometrial carcinoma before menopause is generally more accurate than after menopause. Given that more than 80% of perimenopausal women with endometrial cancer are diagnosed with abnormal uterine bleeding lasting over a month, we recommend conducting endometrial biopsies for such individuals. The menstrual irregularities we have identified may pose a high risk for the development of endometrial carcinoma, rather than serving as a diagnostic indicator.

## Figures and Tables

**Table 1:** Differences in abnormal uterine bleeding in menopausal endometrial cancer patients.

	Premenopause group	Menopause group	*P* value
Case number (*N*)	441	487	
Age	46.49 ± 5.91	58.50 ± 6.86	<0.001
BMI			0.253
<18.5	10 (2.3)	8 (1.6)	
18.5-23.9	178 (40.4)	167 (34.3)	
≥24	253 (57.4)	312 (64.1)	
Gravida (times)			<0.001
0	35 (7.9)	9 (1.8)	
1-2	168 (38.1)	164 (33.7)	
>2	238 (54.0)	314 (64.5)	
Parity (times)			<0.001
0	52 (11.8)	12 (2.5)	
1	233 (52.8)	235 (48.3)	
≥2	156 (35.4)	240 (49.3)	
Menarche age			<0.001
<11	4 (0.9)	1 (0.2)	
11-13	105 (23.8)	64 (13.1)	
≥13	332 (75.3)	422 (86.7)	
Regular cycle(year)	30.55 ± 6.38	34.20 ± 4.01	<0.001
Duration of AUB (month)	14.87 ± 26.84	6.81 ± 11.58	<0.001
Contraceptive way			0.531
IUD	75 (17.0)	72 (14.8)	
Drug	3 (0.7)	3 (0.6)	
Others	26 (5.9)	40 (8.2)	
No	337 (76.4)	316 (64.9)	
Hypertension			<0.001
Yes	61 (13.8)	148 (30.4)	
No	380 (86.2)	339 (69.6)	
Diabetes			0.007
Yes	38 (8.6)	87 (17.8)	
No	403 (91.4)	400 (82.1)	
Malignant tumor history^∗^			0.843
Yes	73 (16.6)	81 (16.6)	
No	368 (83.4)	406 (83.4)	
Degree of differentiation (FIGO)			<0.001
III	34 (7.7)	85 (17.5)	
II	196 (44.4)	253 (52.0)	
I	211 (47.8)	149 (30.6)	
Stage			0.028
I	342 (77.6)	387 (79.5)	
II	50 (11.3)	39 (8.0)	
III	47 (10.7)	49 (10.1)	
IV	2 (0.5)	12 (2.5)	

BMI: body mass index; IUD: intrauterine device; FIGO: Federation International of Gynecology and Obstetrics. ^∗^Family history of malignant tumors includes parents suffering from lung cancer, colon cancer, liver cancer, endometrial cancer, ovarian cancer, and prostate cancer.

**Table 2:** Different analysis of clinical characteristics in endometrial carcinoma patients, categorized based on the duration of abnormal uterine bleeding (AUB).

Duration of AUB (month)	Less than 1 M	1-6 M	7-24 M	More than 24 M	*P* value
Case number	283	314	251	80	
Average age	54.57 ± 9.01	52.37 ± 8.15	52.47 ± 8.54	49.18 ± 9.85	<0.001^1^
Menarche age	13.99 ± 1.90	13.83 ± 1.86	13.89 ± 1.89	13.75 ± 1.71	0.682^1^
Menopause					<0.001^2^
No	95	156	134	56	
Yes	188	158	117	24	
Age	49.89 ± 3.78	50.01 ± 3.29	49.70 ± 3.44	50.25 ± 2.66	0.857^1^
Gravida (times)	3.11 ± 1.81	3.04 ± 1.64	2.98 ± 1.77	3.15 ± 2.10	0.808^1^
Parity (times)	1.58 ± 1.04	1.50 ± 0.89	1.54 ± 1.02	1.51 ± 1.43	0.864^1^
Degree of differentiation (FIGO)					0.266^2^
I	94	129	107	30	
II	145	146	116	42	
III	44	39	28	8	
Stage					0.086^2^
I	218	253	191	67	
II	21	36	26	6	
III	37	24	30	5	
IV	7	1	4	2	

AUB: abnormal uterine bleeding; FIGO: Federation International of Gynecology and Obstetrics.

## Data Availability

No data is available.
